# A Novel Mutation in the *INSR* Gene Causes Severe Insulin Resistance and Rabson–Mendenhall Syndrome in a Paraguayan Patient

**DOI:** 10.3390/ijms25063143

**Published:** 2024-03-08

**Authors:** Maria Natalia Rojas Velazquez, Fabiola Blanco, Ana Ayala-Lugo, Lady Franco, Valerie Jolly, Denisse Di Tore, Idoia Martínez de Lapiscina, Marco Janner, Christa E. Flück, Amit V. Pandey

**Affiliations:** 1 Department of Pediatrics, University Children’s Hospital Bern, Inselspital, 3010 Bern, Switzerland; maria.rojasvelazquez@students.unibe.ch (M.N.R.V.); marco.janner@insel.ch (M.J.); christa.flueck@unibe.ch (C.E.F.); 2 Translational Hormone Research Program, Department of Biomedical Research, University of Bern, 3010 Bern, Switzerland; 3 Graduate School for Cellular and Biomedical Sciences, University of Bern, 3010 Bern, Switzerland; 4 Facultad de Ciencias Médicas, Universidad Nacional de Asunción, Asunción 111241, Paraguay; fabiola.blanko@gmail.com; 5 Instituto de Previsión Social, Asunción 172755, Paraguay; 6 Laboratorio de Genética Molecular, Departamento de Genética, Instituto de Investigaciones en Ciencias de la Salud, Universidad Nacional de Asunción, Asunción 111241, Paraguay; anaayalalugo@gmail.com (A.A.-L.); ladyfran007@gmail.com (L.F.); vali.jolly@gmail.com (V.J.); denisseditore@gmail.com (D.D.T.)

**Keywords:** Rabson–Mendenhall syndrome, insulin resistance, diabetes mellitus, acanthosis nigricans, tyrosine kinase, insulin, insulin receptor, INSR

## Abstract

Rabson–Mendenhall syndrome (RMS) is a rare autosomal recessive disorder characterized by severe insulin resistance, resulting in early-onset diabetes mellitus. We report the first case of RMS in a Paraguayan patient. The patient is a 6-year-old girl who presented with hypertrichosis, acanthosis nigricans, nephrocalcinosis, and elevated levels of glucose and insulin that served as diagnostic indicators for RMS. Genetic testing by next-generation sequencing (NGS) revealed two pathogenic variants in exons 2 and 19 of the *INSR* gene: c.332G>T (p.Gly111Val) and c.3485C>T (p.Ala1162Val), in combined heterozygosis. The novel *INSR* c. 332G>T variant leads to the substitution of glycine to valine at position 111 in the protein, and multiple in silico software programs predicted it as pathogenic. The c.3485C>T variant leads to the substitution of alanine to valine at position 1162 in the protein previously described for insulin resistance and RMS. The management of RMS is particularly challenging in children, and the use of metformin is often limited by its side effects. The patient was managed with nutritional measures due to the early age of onset. This report expands the knowledge of RMS to the Paraguayan population and adds a novel pathogenic variant to the existing literature.

## 1. Introduction

The insulin receptor is a tyrosine kinase that guides the action of insulin. It is encoded by a single gene called *INSR* located on chromosome 19 (19p13.2, genomic coordinates (GRCh38): 19:7,112,265-7,294,414) which has 22 exons. The INSR protein exists as a tetramer of two alpha and two beta subunits linked by disulfide bonds. The binding of insulin molecules to INSR activates the kinase function leading to the phosphorylation of multiple downstream substrates, which mediate the actions of insulin in human metabolism. Mutations in the insulin receptor gene (*INSR*, MIM: *147670) are the root cause of multiple genetic disorders [1,2,3,4] including RMS (MIM: #262190), which is a rare autosomal recessive genetic disorder [5,6,7] that affects the endocrine, metabolic, and immune systems.

Classic features of RMS include acanthosis nigricans (MIM: %100600), a skin condition that causes dark, velvety patches; dental abnormalities; growth retardation; and other systemic abnormalities [8,9,10,11]. Robert Rabson and Edwin Mendenhall reported two siblings with features of diabetes mellitus, acanthosis nigricans, and abnormal dentition in 1956, when the disorder was described for the first time [8]. From that point forward, only a few cases of RMS have been accounted for around the world, and limited reports exist about the clinical and hereditary elements of the issue [2,5,6,10,12]. RMS can appear in a variety of ways, and the severity of the condition can vary from person to person [1,9,13]. While some patients present with milder forms of the disorder, others may have more severe manifestations such as early-onset diabetes, severe insulin resistance, and recurrent infections [12,14,15]. It can be difficult to make a diagnosis because RMS’s clinical features may also overlap with those of other metabolic and genetic disorders [16,17,18].

Clinical features, such as the presence of acanthosis nigricans, insulin resistance, and dental abnormalities, as well as laboratory tests that confirm hyperglycemia and hyperinsulinemia, are used to make the diagnosis of RMS [15,19,20]. The diagnosis is confirmed through genetic testing and the identification of a specific variant in the *INSR* gene [21,22]. Crucially, managing blood glucose emerges as a dominant concern, especially in the initial stages marked by significant fluctuations between fasting hypoglycemia and severe postprandial hyperglycemia. While insulin therapy proves limited in certain cases, prioritizing blood glucose control is paramount. Dyslipidemia and hypertension, typically less pronounced in younger patients with severe defects, become more pertinent in older individuals with higher residual INSR function [9,10,14,18]. Understanding the genetic basis of the disease guides effective management strategies for RMS, underscoring the significance of tailored approaches in addressing its complex metabolic components [13,17].

## 2. Results

**Figure 1:**
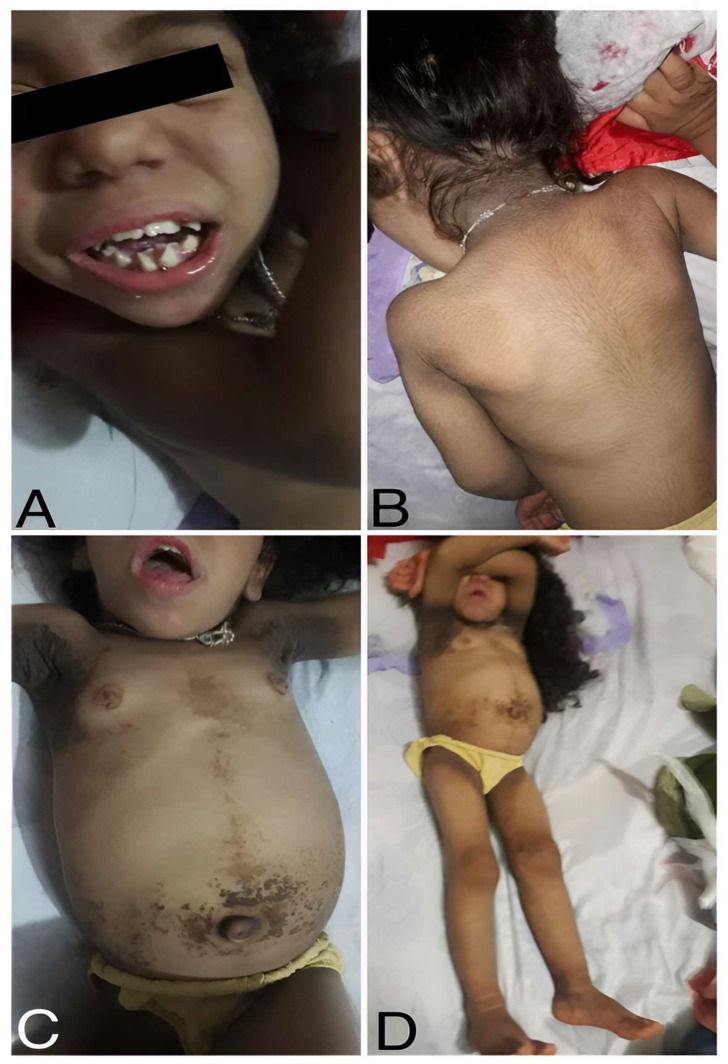
Clinical presentation of the patient (4 years old at the time when pictures were taken). (**A**) Acromegalic facies with enlarged facial features and an ogival arch palate. (**B**) Rough skin, dry hair, and generalized hypertrichosis (excessive hair growth). (**C**) Umbilical and epigastric hernia, along with a globular, distended abdomen. (**D**) Acanthosis nigricans are visible in the neck, axilla, belly, and inner thighs. All the pictures were taken in March 2021.

A 6-year-old girl (born in February 2017) from Colonel Oviedo, a rural area in Paraguay, first visited the clinic when she was 8 months old for a consultation due to suspected early puberty. Upon physical examination, the girl exhibited distinctive facial features, including acromegalic facies and an ogival high arch palate (Figure 1). Additionally, she displayed hyperkeratosis, rough skin, dry hair, and generalized hypertrichosis, indicating excessive hair growth across her body (Figure 1). She had acanthosis nigricans in the neck, in the axilla, around the abdomen, and on the inner surface of both thighs in the upper third. She also had breast enlargement up to M3. The clitoris was hypertrophied, and the pubic hairs were hyperpigmented but not curly (Tanner 2). Crying revealed an umbilical and epigastric hernia, as well as a globular, distended abdomen without palpable visceromegaly (Figure 1).

Biochemical laboratory values at the first visit revealed high levels of glucose and insulin in the blood (Table 1), indicating insulin resistance syndrome. The parent had no consanguinity or familial history of diabetes. Two older siblings (an 11-year-old boy and a 7-year-old girl) were reported as healthy. In the beginning, nutritional measurements were used to treat the patient. Dietary management predominantly focused on a low-carbohydrate regimen comprising dairy, fruits, vegetables, and meat, while omitting complex carbohydrates like flours. Home glucose monitoring was undertaken; plasma glucose variations unveiled fasting levels around 5 mmol/L following a low-carb dinner. However, postprandial glucose surged to 11–22 mmol/L. Therefore, the patient initiated metformin treatment in 2018 with an initial dosage of 100 mg thrice daily; the treatment was discontinued for some time due to the early age of the patient [9,17,23].

The patient exhibited clinical stability under dietary intervention until the year 2020, at which point a respiratory tract infection, potentially attributable to COVID-19, precipitated a deterioration in the patient’s clinical state, metabolic parameters, and the severity of acanthosis. Therapeutic interventions subsequently employed encompassed dietary modification through fruit restriction, heightened medical surveillance, reinstatement of metformin therapy 850 mg twice daily, and a directed referral for genetic testing.

**Figure 2:**
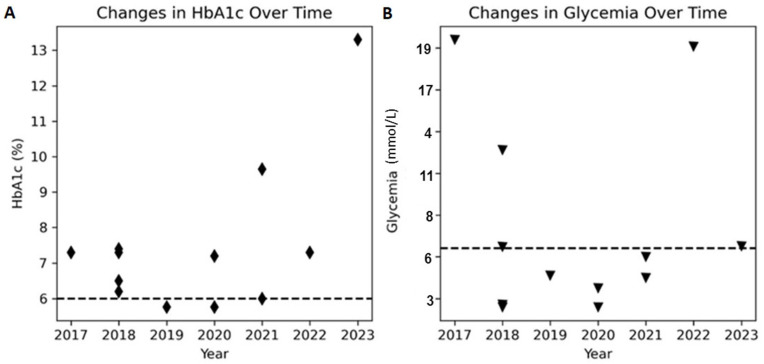
Longitudinal changes in HbA1c and glycemia levels over time in the patient. The plot shows (**A**) the trend of HbA1c values as black diamond markers and (**B**) glycemia levels as black triangle markers. In each panel, a horizontal black dashed line indicates the maximum normal range for either HbA1c or glycemia. The figure shows the fluctuation of HbA1c and glycemia levels over time, highlighting the challenges in managing glucose control in the patient’s condition.

Figure 2 illustrates the longitudinal changes in HbA1c and glycemia levels across multiple visits to the physician. The plot displays the respective trend of HbA1c values and plasma glucose levels. The figure visually shows the fluctuations in HbA1c and plasma glucose levels over time. The patient commenced prandial insulin in June 2021, followed by the introduction of basal insulin in July 2022. Presently, despite these therapeutic measures, intermittent postprandial glucose levels exceeding 22 mmol/L persist.

Prandial insulin constitutes fast-acting insulin administered before meals to mitigate postprandial glucose excursions, aligning with the surge in blood sugar associated with food intake. In contrast, basal insulin provides a gradual and sustained release throughout the day, acting as the foundational insulin requirement to sustain glucose levels between meals and during fasting periods. The combination of prandial and basal insulin is frequently employed to achieve comprehensive glycemic control, especially when dietary modifications and other medications prove insufficient. Despite these interventions, the ongoing complexities in regulating postprandial glucose emphasize the nuanced nature of the patient’s metabolic condition.

**Figure 3:**
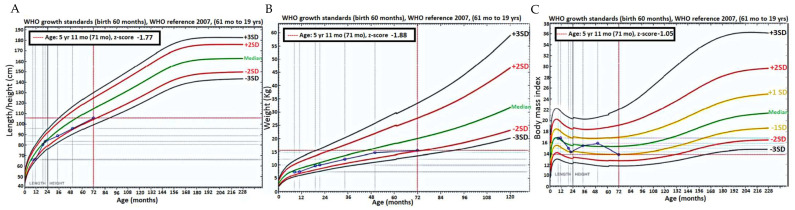
World Health Organization (WHO) child growth standards. (**A**) Height curve. (**B**) Weight curve. (**C**) BMI curve. The patient data are represented in blue. In each panel, the percentiles are shown in different colors. Black Curves: Indicate deviations of ±3 Standard Deviations (SD) from the mean. Red Curves: Represent deviations of ±2 SD from the mean. Yellow Curves: Denote deviations of ±1 SD from the mean. The Z-scores, which quantify the number of standard deviations a data point is from the mean of a reference population, are marked by intersections with intermittent red lines.

At the initial visit, at 8 months of age, the patient showed a length of 66 cm (−2.0 SDS) followed by a transitory catch-up growth at 20 months and subsequent faltering of her growth and height at ages 4 and 6 years at −2.0 SDS (Figure 3A). The patient always showed an appropriate weight gain up to age 4 years. Between 4 and 6 years of age, she only gained 0.9 kg of weight (Figure 3B). However, the body mass index (BMI) remained in the normal range at 13.8 kg/m^2^ (−1.0 SDS) (Figure 3C).

In summary, her acromegalic facies, ogival palate, hyperkeratosis, rough skin, dry hair, generalized hypertrichosis, breast enlargement, and clitoris hypertrophy are suggestive of RMS [11,15,19].

**Figure 4:**
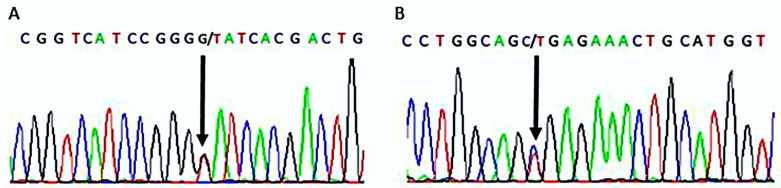
Sanger sequencing confirmation of combined heterozygous mutations of *INSR*. (**A**) Heterozygous mutation of *INSR* Chr19:7267665 (GRCh38), c.332G>T, p.Gly111Val. (**B**) Heterozygous mutation of *INSR* Chr19:7122658 (GRCh38), c.3485C>T, p. Ala1162Val.

The patient in this case report presented with a constellation of clinical features consistent with RMS. Genetic testing confirmed the suspected diagnosis, revealing two pathogenic variants in the *INSR* gene. Specifically, the patient presented as a compound heterozygote carrying the c.332G>T (p.Gly111Val) located in exon 2 (Figure 4A) and with c.3485C>T (p.Ala1162Val) located in exon 19 (Figure 4B) in a combined heterozygous state in the *INSR* gene.

The identified variants p.Gly111Val and p.Ala1162Val were analyzed using several different prediction tools, each offering valuable insights into the potential impact of amino acid changes caused by mutations. These tools are useful in genetic analysis, as they help researchers predict whether a genetic variant is likely to be harmful or benign.

First, Sorting Intolerant From Tolerant (SIFT, https://sift.bii.a-star.edu.sg/) [24] predicted both variants as deleterious, suggesting they have low tolerance within protein structures (Table 2). The term low tolerance suggests that these variants may lead to functional disruptions or structural instability, potentially causing adverse effects on protein function. Protein Variation Effect Analyzer (PROVEAN, http://provean.jcvi.org/index.php) [25] also classified both mutations as deleterious, indicating a significant potential impact on protein function. Additionally, Polymorphism Phenotyping v2 (PolyPhen-2, http://genetics.bwh.harvard.edu/pph2/) [26] indicated a high probability of the mutations being damaging.

Furthermore, Protein Annotation Through Evolutionary Relationship (PANTHER, https://www.pantherdb.org/tools/) [27] classified the variants as ‘Probably damaging’, further underscoring the potential for deleterious effects. MutationTaster2021 [28] labeled them as disease-causing (D), indicating a high likelihood of pathogenicity. Combined Annotation-Dependent Depletion (CADD, https://cadd.gs.washington.edu/snv) [29] categorized the variants as pathogenic based on their scores.

Moreover, Single Nucleotide Polymorphism and Gene Ontology (SNPs&GO, https://snps.biofold.org/snps-and-go/) [30] and MutPred2 [31] predictions associated the variants with the disease, providing additional support for their pathogenic nature. The comprehensive analysis and interpretation of prediction scores can be found in Table 2. Taken together, these findings strongly support our hypothesis that the variants p.Gly111Val and p.Ala1162Val in INSR play a pivotal role in the observed RMS phenotype exhibited by the patient.

*INSR* c.332G>T leads to the substitution of glycine for valine at position 111 in the protein. Although this variant has not been previously described in the literature, several prediction software programs (SIFT [24], PROVEAN [25], Polyphen-2 [26], PANTHER [27], MutationTaster2021 [28], CADD [29], SNPs&GO [30], and MutPred2 [31]) predicted it as pathogenic. Further functional studies would be necessary to confirm the pathogenicity of this variant.

**Figure 5:**
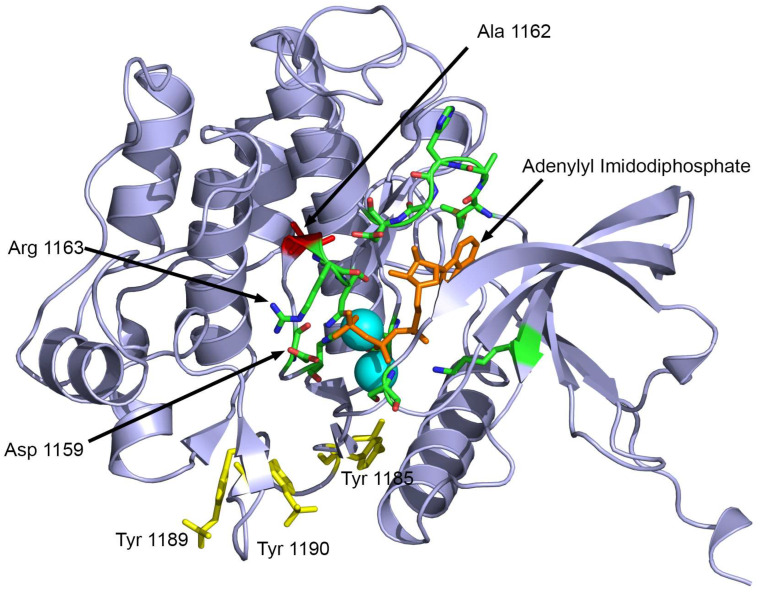
Structural analysis of the Ala1162Val mutation in INSR. The alanine-to-valine substitution at position 1162 is near the catalytic center (Asp 1159) of the tyrosine kinase domain of the INSR. Structure features are illustrated using the X-ray crystal structure of the tyrosine kinase domain of INSR (PDB: 1IR3). The ATP analog adenylyl imidodiphosphate is shown as orange sticks, and Mg^+^ ions are shown as cyan spheres. The alanine 1162 is shown in red while active site residues are shown in green. The phosphorylated tyrosine residues in activated INSR are shown as yellow sticks.

*INSR* c.3485C>T, on the other hand, leads to the substitution of alanine for valine at position 1162 in the protein. This variant has been previously described in the literature in cases of insulin resistance and RMS [32]. Another study reported a different change in the same residue, where glutamic acid was substituted for alanine 1135 (a different isoform was used as a reference sequence; therefore, there is a difference in the numbers reported) in the putative “catalytic loop” of the tyrosine kinase domain of the human insulin receptor [33]. The alanine 1162 is located close to residues involved in ATP binding (1163–1167) and the active site (1159) of INSR (Figure 5). The mutation Ala1162Val potentially impairs the receptor tyrosine kinase activity of INSR due to disrupted ATP binding.

## 3. Discussion

RMS is a rare genetic disorder characterized by severe insulin resistance, resulting in diabetes mellitus, hypertrichosis, acanthosis nigricans, and nephrocalcinosis [18,22,34]. Early diagnosis and management are crucial to prevent complications and optimize the patient’s outcome and quality of life [17]. This case highlights the challenges in managing the complexity of RMS in a low-income setting [9]. The rarity of this disease and the lack of awareness among healthcare professionals often lead to delayed diagnosis and inappropriate treatment [11,35]. Moreover, genetic diagnosis in low-income families in Paraguay is particularly difficult. This is the first report of RMS in the Paraguayan population, where genetic testing is not readily available. However, genetic testing is essential for the accurate diagnosis and management of this disease [21], as it can identify pathogenic variants in the *INSR* gene, such as c.332G>T and c.3485C>T, which were found in this patient. The exact mechanisms of pathogenic effects of mutations in the *INSR* gene seem to be variable and include disrupted membrane localization [36,37], reduced binding to insulin [38], and impairment of proteolytic processing and transport to the cell surface [33].

The management of the presented case involves a multifaceted approach aimed at addressing the complexities associated with RMS and its impact on glycemic control [4,12,38]. The initial reliance on dietary treatments underscores the importance of nutritional interventions in mitigating the metabolic challenges posed by RMS. Later on, metformin treatment was also implemented. However, the exacerbation of the patient’s condition following a respiratory tract infection in 2020, potentially related to COVID-19, necessitated a reevaluation of the therapeutic strategy. The subsequent inclusion of prandial insulin in June 2021 and basal insulin in July 2022 reflects a progressive shift towards a more intensive insulin regimen to manage glycemic fluctuations. Despite these interventions, the persistence of postprandial glucose levels exceeding 300 mg/dL indicates the ongoing intricacies in achieving optimal glycemic control in this unique clinical scenario.

The challenges in glycemic management also highlight the need for ongoing monitoring, close clinical follow-up, and a personalized treatment approach tailored to the patient’s evolving clinical course. The combination of prandial and basal insulin is a well-established strategy in diabetes management; however, its effectiveness in the context of RMS requires continuous evaluation. Further adjustments in insulin dosages or consideration of additional therapeutic modalities may be warranted to address persistent postprandial hyperglycemia. Additionally, the recurrent nature of glycemic fluctuations underscores the importance of continuous patient education and engagement to enhance adherence to treatment plans [17,23]. The nephrocalcinosis observed in this case is a known complication of RMS and reflects the underlying metabolic disruption [16]. Close monitoring of renal function is essential in these patients, and regular imaging studies are required to detect any changes in the renal structure [14,16]. The hypertrichosis and acanthosis nigricans are also typical features of this syndrome and reflect the underlying insulin resistance. The cosmetic impact of these skin changes should not be underestimated, as they can significantly affect the quality of life of affected individuals [5,8].

The child showed poor linear growth and insufficient weight gain between the ages of 4 and 6 years. These findings emphasize the need for comprehensive evaluation and management of the patient’s growth. Close monitoring, multidisciplinary intervention, and early interventions such as nutritional supplementation are crucial to optimize the patient’s growth trajectory and prevent potential long-term complications. Regular follow-up and adjustments to the management plan are necessary to track progress and enhance the patient’s overall growth and development [8,11,20].

Among the treatments that could be considered for future treatment is the usage of metreleptin, a recombinant analog of human leptin, which has been approved for the treatment of generalized lipodystrophy, a rare metabolic disorder characterized by the loss of adipose tissue [20,23,39]. Although there are no clinical trials evaluating the efficacy of metreleptin in the treatment of RMS, some case reports have suggested that it may improve insulin resistance and hyperglycemia in patients with this condition [20,32]. However, the high cost of metreleptin therapy presents a significant barrier to access, particularly in low-income countries such as Paraguay. Therefore, while metreleptin may hold promise as a potential therapy for RMS, its use is currently limited by economic factors and availability in this setting [9,21].

This case calls attention to the need for a multidisciplinary approach to the management of RMS. Regular follow-up visits and close monitoring of metabolic and renal function are essential to prevent complications and optimize the patient’s outcome. This case report emphasizes the importance of genetic testing in the diagnosis and management of rare genetic disorders such as RMS, especially in low-income settings. Early recognition and appropriate management can significantly improve the patient’s outcome and quality of life.

## 4. Materials and Methods

### 4.1. Sample Preparation and DNA Extraction

Blood samples were collected from the patient after obtaining written informed consent from the parents. Extraction of DNA from peripheral blood leukocytes was performed using the Wizard Genomic DNA purification Kit (Promega, Madison, WI, USA), following the manufacturer’s protocol.

### 4.2. Whole-Exome Sequencing (WES) and Bioinformatic Analysis

WES was performed by Novogene (Novogene Company Limited, Cambridge, UK). Libraries were prepared with a SureSelect Human All Exon V6 capture kit (Agilent, Santa Clara, CA, USA) and sequenced with the NovaSeq 6000 platform (Illumina, San Diego, CA, USA). The genome dataset was aligned with human genome GRCh38 and annotated with wANNOVAR [40]. Variants were filtered by a disorders of sex development (DSD)-related gene list [41] and to identify rare variants with a minor allele frequency (MAF) of <1% in publicly available databases (e.g., dbSNP, gnomAD, accessed on 2 August 2023), using R software (R 4.2.0). The possible effects of identified nonsynonymous genetic variants on the structure and function of the protein were assessed using SIFT (accessed on 24 August 2023) [24], PROVEAN (accessed on 24 August 2023) [25], Polyphen-2 (accessed on 28 August 2023) [26], PANTHER (accessed on 28 August 2023) [27], MutationTaster2021 (accessed on 24 August 2023) [28], CADD (accessed on 25 August 2023) [29], SNPs&GO (accessed on 24 August 2023) [30], and MutPred2 (accessed on 29 August 2023) [31]. Variants were classified for pathogenicity according to the standards and guidelines of the American College of Medical Genetics and Genomics (ACMG) [42].

Structural analysis of disease-causing variant Ala1162Val was performed using the known X-ray crystal structure of the INSR tyrosine kinase domain (PDB: 1IR3) in complex with an ATP analog [43] using Pymol v. 1.5.0.1 (www.pymol.org) and Yasara structure v. 21.12.19 (www.yasara.org) [44].

### 4.3. Variant Validation

The variants identified by WES were validated by Sanger sequencing (Supplementary Materials). Primers were designed using SNAPGene and PCR was performed using Taq polymerase with the following primers: For sequencing p.Gly111Val, the forward primer was 5′-ACGAGGCCCGAAGATTTCC-3′ and the reverse primer was 5′-CCCCGGGTGATGTTCATCAG-3′. For sequencing p.Ala1162Val, the forward primer was 5′-CACCAACCCCGTGTTTCTG-3′, and the reverse primer was 5′-CCTGGCCTGGGTCGTTATG-3′. The PCR products were then sequenced in Macrogen Inc. (Seoul, Republic of Korea) and the data obtained were analyzed using BioEdit v. 7.2.5.

## Figures and Tables

**Table 1:** Biochemical laboratory data from the first consultation at 8 months old. HbA1c, glycosylated hemoglobin; LH, luteinizing hormone; FSH, follicle-stimulating hormone; 17-OHP, 17-hydroxyprogesterone.

Parameters	Units	Values	Normal Range
Plasma glucose	mmol/dL	17	3–6
Insulin	pmol/L	15,539	36–126
HbA1c	%	7.3	4.6-6
LH	IU/L	0.00	<0.02–18.3
FSH	mIU/L	0.55	0–5.0
Estradiol	pg/mL	<11	6–27
Total Testosterone	nmol/L	0.0007	<0.3
Androstenedione	nmol/L	0.01	0.2–0.6
17-OHP	nmol/L	0.1	<3.03
Cortisol in serum	nmol/L	275.9	(AM) 124–662
Urea	mmol/L	9.3	1.7–8.3
Creatinine	μmol/L	16.8	20–66
Sodium	mmol/L	138	133–145
Potassium	mmol/L	4.5	3.1–5.4
Calcium	mmol/L	1.31	1.1–1.3

**Table 2:** Prediction analysis scores and interpretation of INSR variants identified in patient.

Tool	p.Gly111Val	p.Ala1162Val	Interpretation
SIFT	Disease (score 0)	Disease (score 0.001)	Benign (score > 0.05) Disease (score ≤ 0.05)
PROVEAN	Deleterious (score −7.6)	Deleterious (score −3.5)	Neutral (score > −2.5) Deleterious (score ≤ −2.5)
PolyPhen-2	Probably damaging (score 1)	Probably damaging (score 0.99)	Probably damaging (score ≥ 0.8) Benign (score < 0.8)
PANTHER	Probably damaging (deleterious 0.85)	Probably damaging (deleterious 0.85)	Probably damaging (deleterious ≥ 0.5) Benign (deleterious < 0.5)
MutationTaster2021	D	D	D (Disease) N (Neutral)
CADD	Deleterious (score 27.3)	Deleterious (score 29)	Deleterious (score ≥ 20) Benign (score < 20)
SNPs&GO	Disease (score 0.7)	Disease (score 0.65)	Disease (score > 0.05) Benign (score ≤ 0.05)
MutPred2	Disease (score 0.95)	Disease (score 0.90)	Pathogenic (score ≥ 0.5) Benign (score < 0.5)

## Data Availability

All data are provided in the paper, except whole-exome sequencing data of the patient, which are not provided due to privacy concerns.

## Supplementary Materials

The supporting information can be downloaded at: https://www.mdpi.com/article/10.3390/ijms25063143/s1.
