# Supplementary material for: A Novel Mutation in the INSR Gene Causes Severe Insulin Resistance and Rabson–Mendenhall Syndrome in a Paraguayan Patient
Source: Int J Mol Sci. 2024 Mar 8;25(6):3143. doi: 10.3390/ijms25063143 (PMC10970221; doi:10.3390/ijms25063143)
Supplement: Supplementary file 1 [file ijms-25-03143-s001.zip › ijms-2733429R4-suplemmentarymaterial.pdf]

SANGER SEQUENCING FOR VALIDATION OF VARIANTS

	Primer sense 5'to 3'	Primer size (bp)	Tm	%GC	Amplicon size	Sequence
**KNOWN VARIANT Chr19:7122658 (GRCh38), c.C3485T, p.A1162V**						
1VRsnap F	CACCAACCCCGTGTTTCTG	19	58	58	230	A
2VRsnap R	CCTGGCCTGGGTCGTTATG	19	59	63		
**NOVEL VARIANT Chr19:7267665 (GRCh38), c.G332T, p.G111V**						
5VNRSNAP F	ACGAGGCCCGAAGATTTCC	19	59	58	224	C
6VNRSNAP R	CCCCGGGTGATGTTCATCAG	19	59	60		

SECUENCE OF KNOWN VARIANT (c.C3485T, p.A1162V) (A)

CACCAACCCCGTGTTTCTGTTTTAGAATAATCCTGGCCGCCCTCCCCCTACCCTTCAAGAGATGATTCAG
ATGGCGGCAGAGATTGCTGACGGGATGGCCTACCTGAACGCCAAGAAGTTTGTGCATCGGGACCTGG
CAGCGAGAAACTGCATGGTCGCCCATGATTTTACTGTCAAAATTGGAGGTTCGTCTGGCTTTCTGCTTT
GAAAACATAACGACCCAGGCCAGG

SECUENCE OF NOVEL VARIANT (c.G332T, p.G111V) (C)

ACGAGGCCCGAAGATTTCCGAGACCTCAGTTTCCCCAAACTCATCATGATCACTGATTACTTGCTGCTCT
TCCGGGTCTATGGGCTCGAGAGCCTGAAGGACCTGTTCCCCAACCTCACGGTCATCCGGGGATCACGA
CTGTTCTTTAACTACGCGCTGGTCATCTTCGAGATGGTTCACCTCAAGGAACTCGGCCTCTACAACCTGA
TGAACATCACCCGGGG

A and C refer to the sequence of each amplicon that are listed. F and R indicate forward and reverse primers.
